# Liposomal delivery of hydrophobic RAMBAs provides good bioavailability and significant enhancement of retinoic acid signalling in neuroblastoma tumour cells

**DOI:** 10.1080/1061186X.2019.1710157

**Published:** 2020-01-14

**Authors:** Maja Bilip, Shreya Shah, Mayuran Mathiyalakan, Aristides D. Tagalakis, Stephen L. Hart, Ruhina Maeshima, Simon Eaton, Michael Orford, Elsa Irving, Alessia Di Florio, Claire Simons, Andrew W. Stoker

**Affiliations:** a Great Ormond Street Institute of Child Health, UCL, London, UK;; b School of Pharmacy and Pharmaceutical Sciences, College of Biomedical and Life Sciences, Cardiff University, Cardiff, UK

**Keywords:** Neuroblastoma, retinoic acid, liposome, RAMBA, CYP26, neural differentiation

## Abstract

Retinoid treatment is employed during residual disease treatment in neuroblastoma, where the aim is to induce neural differentiation or death in tumour cells. However, although therapeutically effective, retinoids have only modest benefits and suffer from poor pharmacokinetic properties. *In vivo*, retinoids induce CYP26 enzyme production in the liver, enhancing their own rapid metabolic clearance, while retinoid resistance in tumour cells themselves is considered to be due in part to increased CYP26 production. Retinoic acid metabolism blocking agents (RAMBAs), which inhibit CYP26 enzymes, can improve retinoic acid (RA) pharmacokinetics in pre-clinical neuroblastoma models. Here, we demonstrate that in cultured neuroblastoma tumour cells, RAMBAs enhance RA action as seen by morphological differentiation, AKT signalling and suppression of MYCN protein. Although active as retinoid enhancers, these RAMBAs are highly hydrophobic and their effective delivery in humans will be very challenging. Here, we demonstrate that such RAMBAs can be loaded efficiently into cationic liposomal particles, where the RAMBAs achieve good bioavailability and activity in cultured tumour cells. This demonstrates the efficacy of RAMBAs in enhancing retinoid signalling in neuroblastoma cells and shows for the first time that liposomal delivery of hydrophobic RAMBAs is a viable approach, providing novel opportunities for their delivery and application in humans.

## Introduction

Neuroblastoma is a paediatric, peripheral nervous system cancer, accounting for 15% of childhood cancer deaths [1] and it presents a stubborn clinical challenge. In aggressive disease, multimodal treatments are followed by maintenance treatment for residual disease, which includes the vitamin A derivative, retinoic acid (RA) [2]. RA induces neuroblastoma tumour cell differentiation or death in a range of neuroblastoma-derived cells in culture [3,4]. Although it is uncertain exactly how RA influences tumour cell behaviour in patients, the treatment does provide modest improvements in event-free survival [2,5]. Nevertheless, the approach of dosing infants and young children with 13-cis retinoic acid (13-cis-RA) is challenging, with sub-optimal exposure [2,6] and dose-limiting toxicities [2,6]. RA action *in vivo* is further hampered by its rapid metabolism by cytochrome P450 enzymes in the liver and the tumour [7]. Chronic induction of P450 enzymes may also underlie a proportion of RA resistance cases [8,9]. Further improvements in RA efficacy *in vivo* are therefore needed and this could be relevant to a range of cancers where retinoids are employed therapeutically [10,11].

In neuroblastoma treatment, 13-cis-RA is viewed as a pro-drug, with its isomer, all-trans RA (ATRA), being the biological effector inside cells [12–14]. ATRA transcriptionally auto-induces P450 enzymes of the CYP26 subclass, triggering its own destruction. These enzymes have thus become druggable targets of interest, since they are largely responsible for RA clearance during treatment [12]. CYP26 inhibitors are known as RA metabolism blocking agents (RAMBAs) and they hold the potential of improving the clinical benefit of retinoid treatments. Numerous RAMBAs exist [15–20] and these can raise the effective intracellular concentrations or serum concentrations of RA [18,21]. One of these, liarozole, decreases the induced loss of ATRA in serum in acute promyelocytic leukaemia patients [22], suggesting a potential approach for suppressing retinoid resistance. Another RAMBA, R116010, is highly CYP26-specific with low IC_50_ and good anti-tumour activity in RA-treated breast cancer models [13,23,24]. R116010 also blocks CYP26 action both in SH-SY5Y neuroblastoma cells and in the liver, leading to increased serum levels of both ATRA and 13-cis-RA in mice [18]. It is possible therefore that RAMBAs could enhance retinoid action in neuroblastoma tumour cells themselves, inducing differentiation, cell death and N-Myc suppression, although this has not been demonstrated directly.

To further improve the pharmacokinetics of retinoids *in vivo*, there is a need for more effective delivery systems for some of the most potent RAMBAs. In this study, we have used RAMBAs based on imidazole (RAMBA C2) and on triazole (RAMBA C17), these having similar, high specificities and *in vitro* EC_50_ values to R116010 [19,20]. C2 and C17, however, are highly hydrophobic compounds and impractical for use in standard oral or intravenous delivery and are therefore not suitable as drugs in this form. Improved delivery and targeting of drugs can nevertheless be achieved using nanotechnology, as evidenced in pre-clinical data using several drugs in neuroblastoma [25–27] and other solid tumours [28]. Liposomes for example have successfully enhanced drug actions by improving stability, serum longevity and therapeutic efficacy [29]. They are suitable for hydrophobic compounds and may also override drug resistance mechanisms [30,31]. Nanoparticles are also known to take advantage of tumour vascular leakiness to more specifically access tumour cells in solid tumours including neuroblastoma [25,28,32]. For RAMBAs, this approach has not been documented to date and we hypothesised that liposomal delivery would improve bioavailability and making such compounds more accessible for use in humans. Our experimental model in cultured tumour cells would thus provide a proof of principle that could potentially augment future application of RAMBA-based therapy *in vivo*.

The objectives in this study were, therefore, to first demonstrate the effectiveness of RAMBAs in enhancing a range of RA actions in neuroblastoma cells, and then to provide a proof of principle that RAMBAs can be effectively delivered to tumour cells using liposomal systems.

## Materials and methods

### Chemicals and antibodies

Chemicals were from Sigma-Aldrich (St. Louis, MO) unless otherwise stated. ATRA and 13-cis-RA (Sigma-Aldrich, St. Louis, MO) were dissolved in either ethanol or DMSO at 10 mM or 50 mM, respectively, and protected from light at –20 °C. RAMBAs methyl 2,2-dimethyl-3-[4-(naphthalen-2-ylamino)phenyl]-3-(1*H*-1,2,4-triazol-1-yl)propanoate (compound 17; C17; molecular weight 400.5) and methyl 3-(1*H*-imidazol-1-yl)-2,2-dimethyl-3-(4-(naphthalen-2-ylamino)phenyl)propanoate (compound 2; C2; molecular weight 399.5) were generated as described [19] and were dissolved in ethanol at 50 mM (C17) and 1 mM (C2; maximal solubility in this solvent). Antibodies were sourced as follows: anti-N-myc (B8.4.B; Insight Biotechnology, Wembley, UK); anti-phospho-Ser472-AKT (ab4060; Cell Signaling Technology, Danvers, MA); anti-AKT (ab9272; Cell Signaling Technology, Danvers, MA); anti-actin (A5316; Sigma-Aldrich, St. Louis, MO); anti-GAPDH (14C10; Cell Signaling Technology, Danvers, MA). HRP-linked secondary antibodies were purchased from DAKO Ltd. (Bucks, UK).

### Cell culture

Human immortalised cell lines SK-N-SH and IMR32 were obtained from ATCC, KELLY/N206 was a gift from Frank Speleman, University of Ghent (STR genotyped), and LAN-5 was from the Children’s Oncology Group Repository (Lubbock, TX). All cells have been validated by short tandem repeat profiling (LGC Standards) and are mycoplasma tested. Cells were maintained at 37 °C, 5% CO_2_. SK-N-SH were cultured in minimum essential medium Eagle, 1% penicillin/streptomycin, 10% foetal bovine serum and 2 mM L-glutamine. LAN-5 and N206 cells were cultured in RPMI 1640 + GlutaMAX^TM^ (Invitrogen, Carlsbad, CA), 10% foetal bovine serum and 1% penicillin/streptomycin, with 25 mM HEPES pH7 (Fisher Scientific, Waltham, MA).

### Immunoblotting

Cells were lysed in 50 mM Tris–Base pH 7.6, 150 mM NaCl, 1% Triton X-100, 1× protease inhibitors, 25 mM sodium fluoride and 1 mM sodium vanadate. Proteins were separated by polyacrylamide electrophoresis and transferred to PVDF (Immobilon-P). Immunodetection was performed using HRP-linked secondary antibodies, and Pierce ECL 2 Substrate (Thermo Scientific, Waltham, MA) and chemiluminescence film (Amersham Hyperfilm ECL, GE Healthcare, Chicago, IL).

### Liposomes

The lipids 1,2-di-O-octadecenyl-3-trimethylammonium propane (DOTMA), 1,2-dioleoyl-*sn*-glycero-3-phosphocholine (DOPC) and cholesterol were purchased from Avanti Polar Lipids (Alabaster, AL). For empty liposomes DOTMA, DOPC and cholesterol were dissolved in chloroform in a final volume of 500 µl at a molar ratio of 37.5%:37.5%:25%. RAMBA C2 and C17 were dissolved in ethanol and added at a 10% molar ratio to lipids DOTMA (35%), DOPC (35%) and cholesterol (20%). Chloroform was rotary evaporated (BÜCHI Labortechnik AG, Flawil, Switzerland) and lipids were rehydrated in nuclease-free water, rotating overnight, to form liposomes at either 1 mg/ml or 2 mg/ml lipids. For the 2 mg/ml mixture, this equates to the equivalent of 1.1 mM DOTMA, 0.11 mM DOPC and 0.64 mM cholesterol, and in these liposomes the RAMBAs were packaged at a maximal potential concentration of 95.7 μg per ml (0.24 mM) in the liposome solution. After synthesis, liposomes were sonicated for 30–45 min, with the aim of achieving a mean size of between 80 and 160 nm. Size and charge were assessed with a Zetasizer Nano ZS (Malvern Panalytical, Worcestershire, UK). Liposomes were stored at 4 °C. To dialyse liposomes, slide-A-Lyzer^TM^ MINI devices (Thermo Fisher Scientific, Waltham, MA; molecular weight 10 kDa; capacity 15 ml) were used with 500 µl liposomes and this was dialysed against 13.5 ml distilled water for 24 h at 4 °C, with one water change.

### Cell viability assays

Cells were plated at 3000 cells per well in 96-well plates. After 24 h, triplicate wells of cells were treated with chemicals and incubated for five or six days, then assayed for cell number using resazurin (R&D Systems, Minneapolis, MN) as per the manufacturer’s guidelines. Reaction product was measured using fluorescence at 540 nm excitation and 590 nm emission wavelengths, in a FLUOstar Optima (BMG Labtech, Aylesbury, UK).

### Neurite outgrowth assays

Cells were treated and either then photographed live or after 4% paraformaldehyde fixation. Five to 10 fields of view were captured under phase contrast per tissue culture well. Neurites greater than one cell body length were measured using the NeuroJ plugin for ImageJ and cell bodies were counted using the ‘point-count’ function. Light microscopy was performed at the Light Microscopy Core Facility, UCL GOS Institute of Child Health. Where statistical analysis was used, a univariate ANOVA analysis was performed using SPSS with 95% confidence intervals. Post hoc tests included a Bonferroni adjustment and a Dunnett test where stated.

### Real-time PCR

LAN5 and SK-N-SH cells were plated at 3 × 10^5^ per well in six-well plates. The next day, cells were treated with 0.1 μM or 0.5 μM ATRA. After 72 h, RNA was extracted and DNAse treated using the TURBO DNA-*free* kit (Ambion, Austin, TX) according to the manufacturer’s recommendations. cDNA was synthesised from 1 µg of total RNA using Transcriptor First Strand cDNA synthesis (Roche, Basel, Switzerland) according to the manufacturer’s recommendations. Real-time PCR was performed using iTaq Universal SYBR Green Supermix (Bio-Rad, Hercules, CA) in triplicate for the CYP26A1 primers (forward: GGC CTT AGG AGC TGT GTA GG; reverse: TTG TCC ACA GGA TAC ACG GT) and a GAPDH control was used for normalisation (forward: ATGACATCAAGAAGGTGGC; reverse: CATACCAGGAAATGAGCTTG). The thermocycling programme was one cycle at 95 °C for 5 min, 95 °C for 15 s, 60 °C for 1 min, followed by 40 cycles of 95 °C for 15 s and 60 °C for 1 min (CFX96 Real-Time System, C1000 Touch Thermal Cycler, Bio-Rad, Hercules, CA). Melt curve analyses were performed from 65 °C to 95 °C, over 5 min. Fold changes in CYP26A1 mRNA were calculated according to the equation: 2^(–△△Ct)^.

### RNAseq analysis

RNA was extracted and 250 ng was processed using Illumina’s TruSeq RNA sample prep kit version 2 (p/n RS-122-2001) according to the manufacturer’s instructions. mRNA was isolated using Oligo dT beads and the purified mRNA was chemically fragmented. cDNA was generated using reverse transcriptase and random primers. Indexing adaptors were ligated and the cDNA libraries were amplified by PCR. Samples were sequenced on a NextSeq 500 (Illumina, San Diego, CA) using a 43 bp paired end run. Data were converted to fastq using Illumina bcl2fastq v2 and aligned to reference using TopHat2 (http://www.genomebiology.com/2013/14/4/R36/abstract). Duplicate reads were removed (Picard v1.100) and transcript abundance estimated with Python package HTSeq (http://dx.doi.org/10.1093/bioinformatics/btu638). Differential expression was determined using DESeq2 (http://doi.org/10.1186/s13059-014-0550-8).

### UV detection of RAMBAs

For standard curve measurement, RAMBAs were dissolved in ethanol and scanned for absorbance from 200 to 450 nm in a UVIKON UV spectrophotometer with Lab Power Junior software. To quantify C2 RAMBA in liposomes, the pre- and post-dialysis mixtures were diluted 1:9 with ethanol and scanned at OD_315_. Background absorbance of the lipids was measured at OD_315_, using empty liposomes similarly dissolved in ethanol.

## Results

### RAMBA induction of CPY26A1 transcription

**Figure 1.**
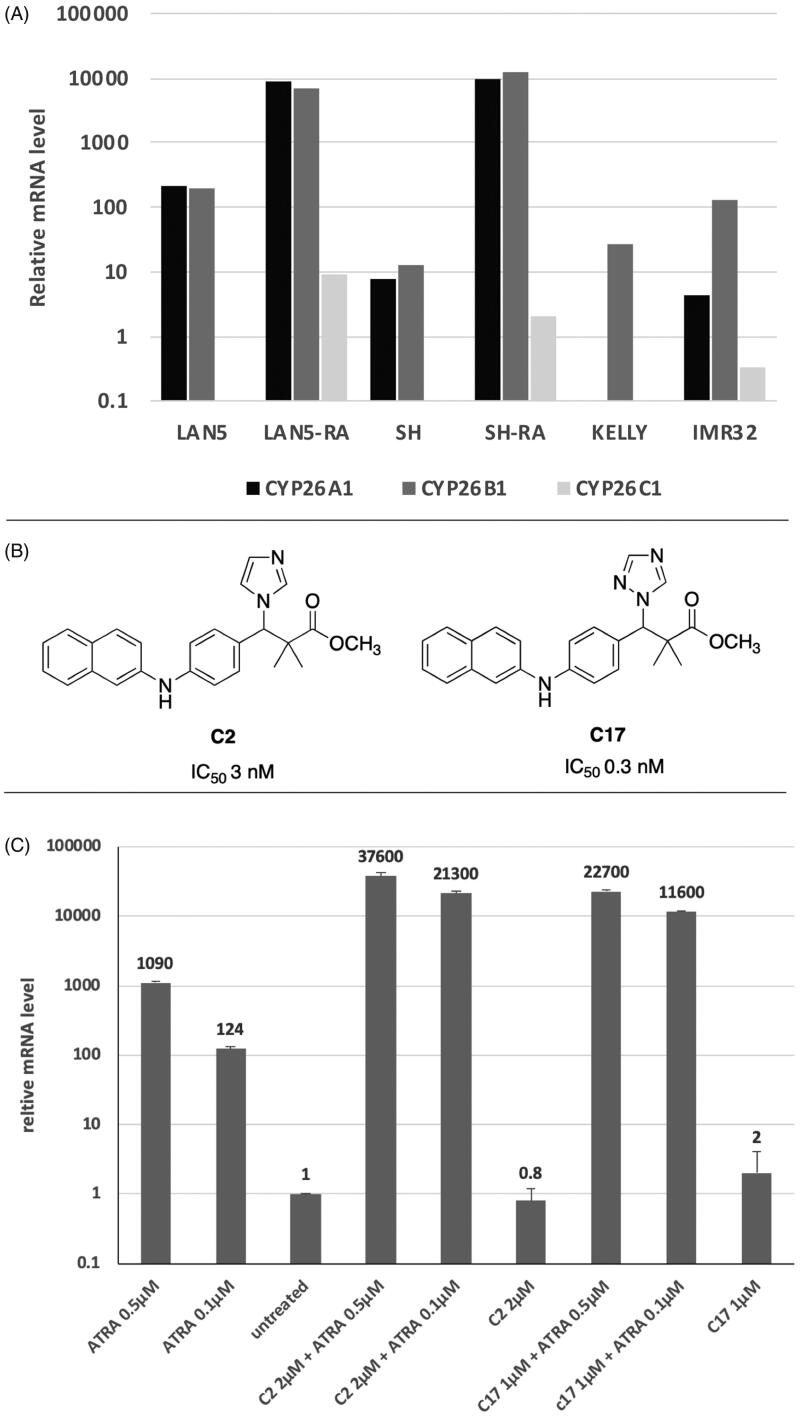
(A) RNAseq analysis of LAN5, SK-N-SH (SH), KELLY and IMR32 cells shows the relative amounts of mRNA present for genes *CYP26A1*, *CYP26B1* and *CYP26C1*. mRNA levels after ATRA treatment for 24 h are also shown for LAN5 and SH cells. (B) Chemical structures of compounds C2 and C17, with their respective IC_50_ values against CYP26A1 [19]. (C) Quantitative PCR was carried out on SK-N-SH cells after treatments with ATRA, RAMBA C2 and C17 and in combinations. Treatments were for 72 h. The relative abundance of *CYP26A1* mRNA is shown, normalised to untreated cells at 1.0. Error bars are standard deviations of triplicate, technical replicates (*n* = 1).

It is unclear what pattern of expression there is of *CYP26* paralogues in neuroblastoma tumour cells. We thus analysed expression of *CYP26A-C* using RNAseq datasets from tumour cell lines LAN5, SK-N-SH, KELLY and IMR32 (Figure 1(A)). LAN5 and SK-N-SH cell samples were either untreated or treated with ATRA (5 µM) for 24 h. KELLY and IMR32 samples were from untreated cells only. *CYP26C* mRNA is not detected except for very low levels in IMR32. *CYP26B1* is expressed in all lines in basal conditions and is the most abundant paralogue in KELLY and IMR32. *CYP26A1* is expressed basally in each cell type except KELLY. *CYP26B1* and *CYP26A1* are strongly induced up to 10,000-fold by ATRA in LAN5 and SK-N-SH. *CYP26C1* is induced by ATRA, but only up to 10-fold, in LAN5 and SK-N-SH cells. In this study, therefore, the RAMBA effects are likely to be predominantly acting through *CYP26A1* and *CYP26B1*.

In our experiments, we used two RAMBA compounds, one based on an imidazole structure (C2) and one on triazole (C17) [19] (Figure 1(B)). C2 and C17 have IC_50_ values of 3 nM and 0.35 nM, respectively, against CYP26 enzymes *in vitro* and can enhance *CYP26A1* mRNA induction by ATRA in SH-SY5Y [19]. To assess RAMBA activity SK-N-SH cells (Figure 1(C)), cells were treated with ATRA at either 0.5 µm or 0.1 µM, with or without the addition of C2 at 2 µM or C17 at 1 µM. When using ethanol-solubilised RAMBAs in SKNSH cells, the control cells contained the equivalent amount of ethanol solvent (less than 0.2% v/v). ATRA stimulated *CYP26A1* transcription up to 1000-fold, whereas the addition of either C2 or C17 induced a 20–30-fold further stimulation of transcription. These data are therefore consistent with previous findings in SH-SY5Y [19], with the RAMBAs strongly enhancing RA’s ability to stimulate *CYP26A1* transcription.

### Stimulation of morphological differentiation by RAMBAs

**Figure 2.**
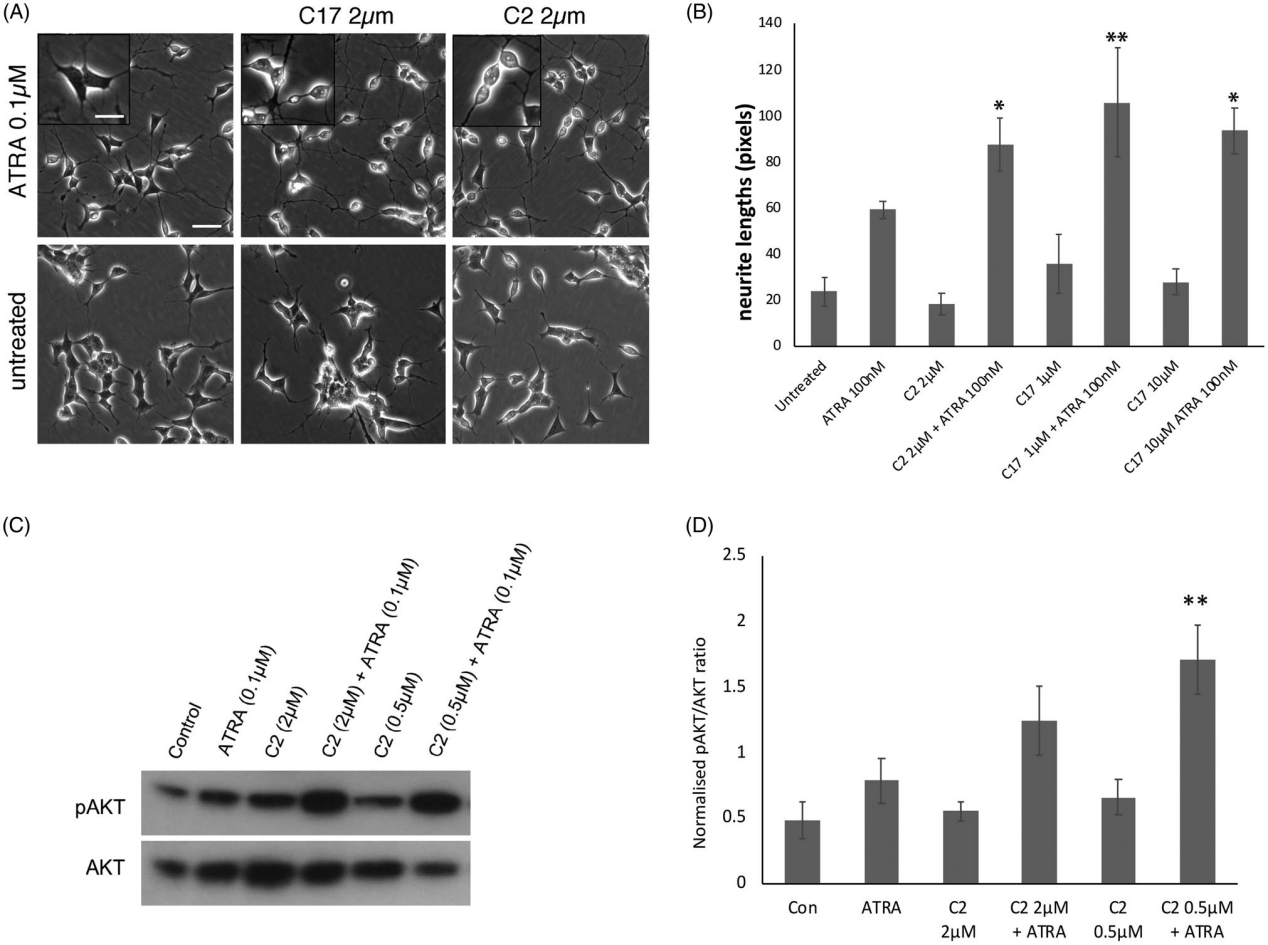
(A) SK-N-SH cells were treated with ATRA alone, or in combination with RAMBA C2 or C17, for 72 h. RAMBAs by themselves had no effect on cell morphology. In combination with ATRA, RAMBAs induce a highly rounded, refractile morphology (insets). Scale bar = 50 µm (25 µm for insets). (B) SK-N-SH cells were treated with 0.1 µM ATRA and RAMBAs C2 and C17 for 72 h and neurite lengths were measured (*n* = 3). Standard deviations are shown. ANOVA comparisons show where mean neurite lengths are significantly longer compared with ATRA alone (Bonferroni’s and Dunnett’s post hoc corrections *p<.05; **p<.01). (C) SK-N-SH cells were treated for 72 h with ATRA, RAMBA C2 and combinations. Immunoblotting shows relative levels of pAKT and AKT present in cell lysates. (D) Protein bands were quantified and mean pAKT intensities were normalised against AKT and compared using ANOVA (*n* = 3). Controls are untreated cells. SD are shown, **p<.01 compared to ATRA alone.

RAMBAs can enhance retinoid-induced differentiation in breast cancer cells [24,33], but the equivalent biological or biochemical responses downstream of RA in neuroblastoma cells have yet to be demonstrated. A key characteristic of retinoid action in neuroblastoma cells is the induction of neuronal differentiation, characterised by highly elongated neurites with growth cones and neuron-like cell bodies [3]. To assess differentiation, C2 and C17 were used alone or in combination with ATRA to treat SK-N-SH cells. Morphological differentiation was gauged by neurite outgrowth and the neuronal cell morphology. In contrast to RAMBAs on breast cancer cells [24,33], RAMBAs C2 and C17 alone had no obvious morphological effect in SK-N-SH (Figure 2(A)). The combination treatments of RAMBAs plus ATRA, however, consistently led to a more extreme morphological differentiation compared with ATRA alone, generating more rounded and phase-bright neurons with extensive processes compared with the more phase dark, polygonal phenotype of ATRA-treated cells (Figure 2(A)). These observations indicate that C2 and C17 can enhance retinoid-stimulation of morphological differentiation. Neurite length quantitation showed that RAMBAs themselves did not affect neurite outgrowth, whereas ATRA treatment did enhance outgrowth (Figure 2(B)). Combination treatments showed that RAMBAs enhanced this ATRA-induced neurite elongation. Increasing the concentration of C17 from 1 µM to 10 µM did not further increase the neurite effect. C2 and C17 RAMBAs can thus enhance the cellular differentiation driven by ATRA in SK-N-SH cells.

### Biochemical enhancement of retinoid action by RAMBAs

Retinoid stimulation of neuroblastoma cells induces a wide range of biochemical changes, including enhancement of AKT phosphorylation, which is required for neurite elongation in ATRA-treated SK-N-SH cells [34,35]. If RAMBAs can enhance the biochemical actions of ATRA, then we should observe a combination effect on AKT phosphorylation. SK-N-SH cells were treated with 0.1 µM ATRA for 72 h in the absence or presence of either 0.5 µM or 2 µM C2. Figure 2(C,D) shows that C2 can indeed enhance the level of pAKT generated by ATRA. This effect was more pronounced with the lower C2 concentration.

### Delivery of RAMBAs using liposomes

So far, we have provided the first demonstration that RAMBAs C2 and C17 can enhance both the biochemical and morphological measures of ATRA signalling in SK-N-SH neuroblastoma cells. C2 and C17 can be delivered to cells after ethanol solubilisation, but their high hydrophobicity would significantly limit their use in humans. A key objective of this work was therefore to assess whether similar RAMBA bioavailability can be facilitated by incorporating these molecules into liposomal nanocomplexes. This would be a proof of principle that nanotechnology could facilitate cellular delivery of such RAMBAs, potentially increasing their therapeutic applicability.

To test liposomal delivery, the C2 and C17 RAMBAs were incorporated into liposomes composed of DOTMA, DOPC and cholesterol (molar ratios 37.5%:37.5%:25%) suspended in water (see Materials and Methods). These kinds of liposome formulations are very effective for cellular delivery [36,37]. After sonication, positively charged liposomes with an average size of 91 nm (C17), 123 nm (C2) and 133 nm (empty) were obtained, with average PDI values from 0.42 to 0.47 (Table 1). We tested only cationic liposomal formulations in order to minimise charge-dependent, non-specific uptake into cultured cells [38]. These liposome solutions would contain, maximally, 240 µM of the RAMBAs and the data below are presented on the basis of this maximal, theoretical incorporation of RAMBAs into the liposomes. The tolerance of neuroblastoma cells for our liposomes allowed us to deliver up to 2 µM of RAMBA without excessive liposomal cytotoxicity. At this dose of RAMBA, a final concentration of 8.3 µg/ml of total lipid would be added to the media. Liposomes were left on cells for the whole treatment period, unless otherwise stated.

**Table 1.** Liposome parameters.

Liposome content	Average size in nm (range)	SD	Average PDI	SD	Average charge (mV)	SD
RAMBA C17 (*n* = 3)	90.9 (82–107)	12.3	0.42	0.02	64.0	7.9
RAMBA C2 (*n* = 6)	123.0 (80–164)	30.4	0.42	0.09	62.5	4.6
Empty (*n* = 6)	133 (100–154)	20.5	0.47	0.14	63.0	5.1

Independent batches of liposomes were made (*n*, number of batches) and analysed in a Zetasizer Nano ZS for size (diameter in nm), polydispersity index (PDI) and average charge (mV). The standard deviation of each set of batches is given.

**Figure 3.**
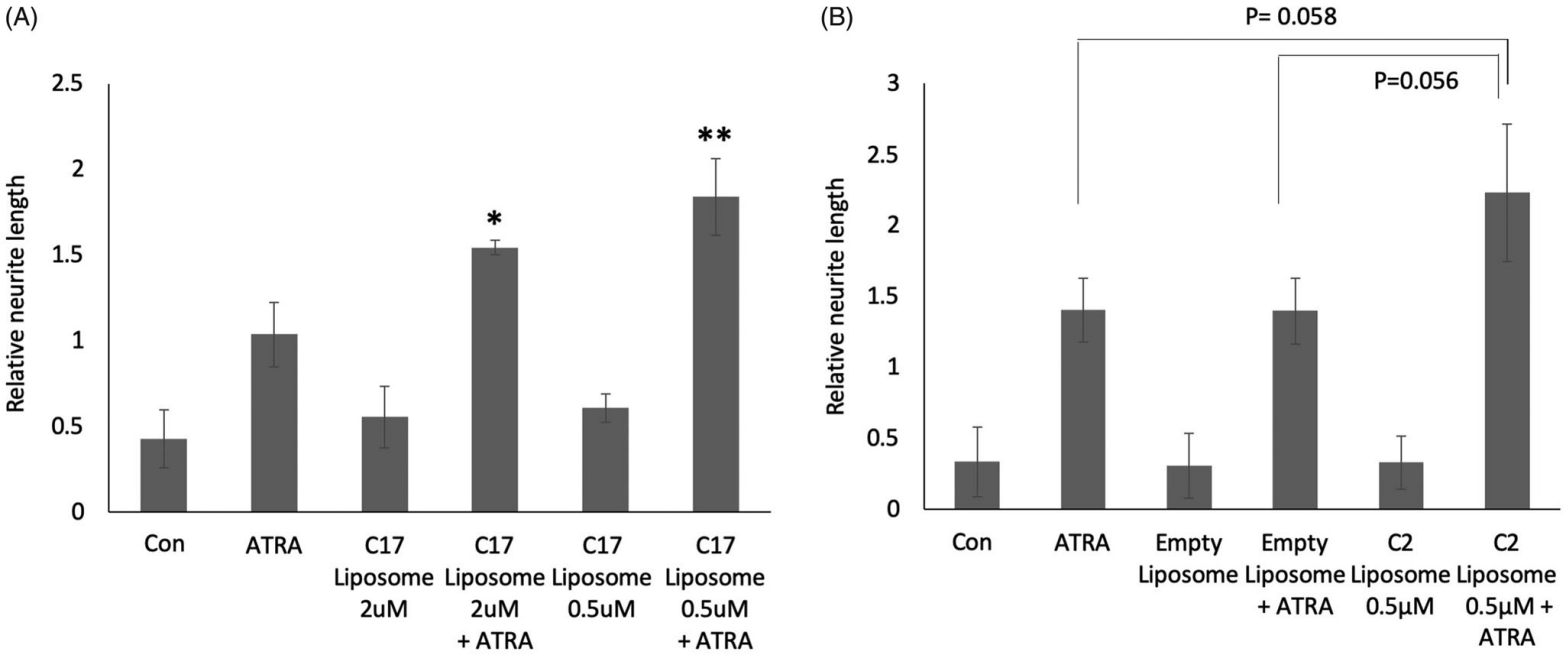
(A) SK-N-SH cells were treated for 72 h with 0.1 µM ATRA, C17-containing liposomes, or combinations of the two. Controls (con) are untreated and maximal concentrations of RAMBAs are indicated. Neurites were measured and statistical comparison made against the mean of ATRA single treatment. SD are shown, *p<.05, **p<.01; *n* = 3. (B) SK-N-SH cells were treated for 72 h with 0.1 µM ATRA, C2-containing liposomes, empty liposomes and combinations (*n* = 3). SD are shown, *p<.05, **p<.01 using ANOVA.

**Figure 4.**
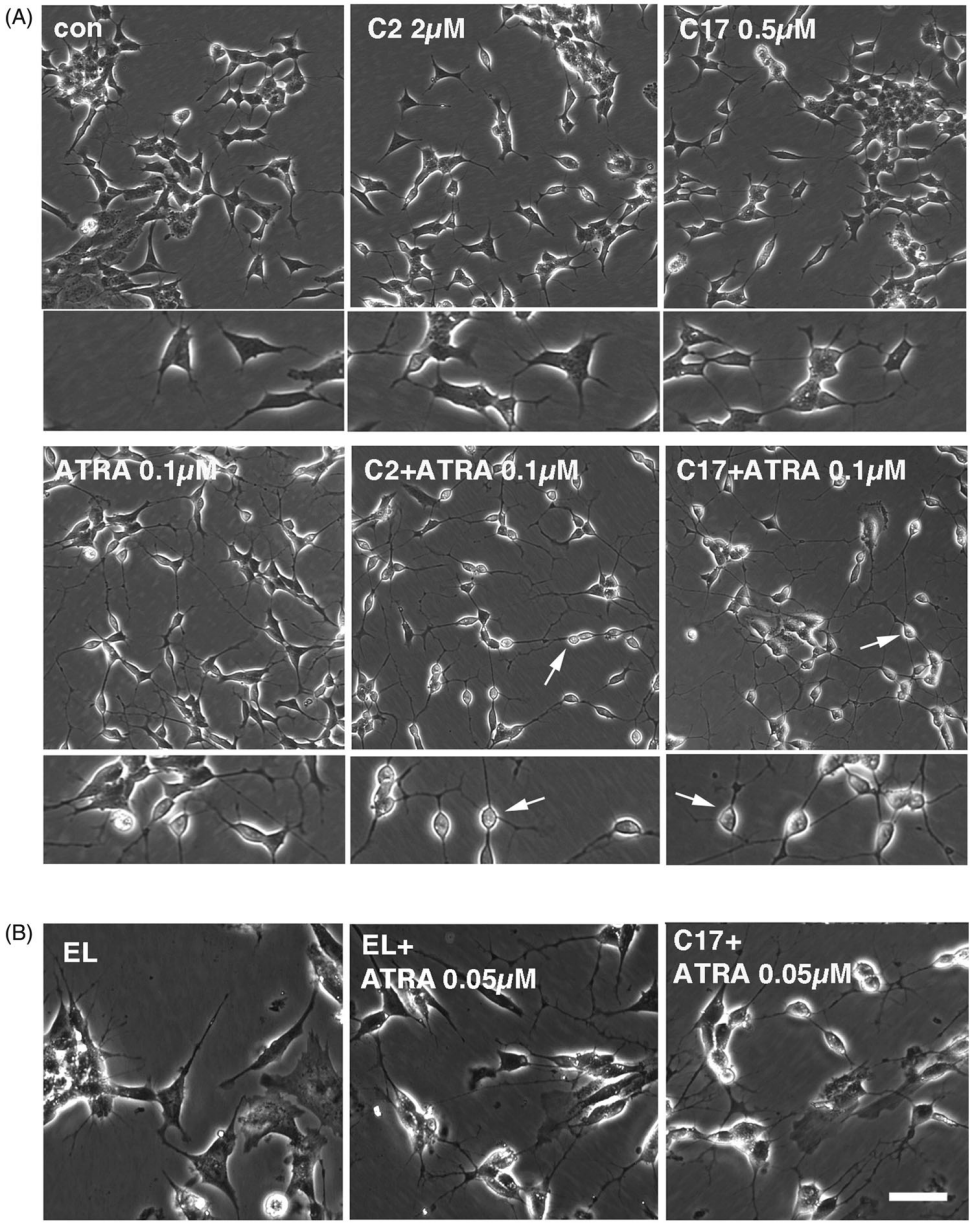
(A) SK-N-SH cells were treated for 72 h with 0.1 µM ATRA, 2 µM C2 liposomes (C2), 0.5 µM C17 liposomes (C17) and combinations thereof. Phase contrast images show that liposomal RAMBAs in combination with ATRA generated highly rounded, neuron-like cells (arrows); 2× enlargements are shown below the main panels. (B) SK-N-SH cells treated for 72 h with empty liposomes (EL) with or without 0.05 µM ATRA, and 0.5 µM C17 liposomes plus 0.05 µM ATRA. Scale bar = 50 µm for rows 2, 4 and 5; 100 µM for rows 1 and 3. Maximal, hypothetical concentrations of the RAMBAs are indicated.

The C17 RAMBA liposomes were initially used to treat SK-N-SH cells, to determine cellular tolerance and enhancement of ATRA-induced differentiation. The data show that liposomal C17 enhances ATRA-induced neurite elongation as effectively as ethanol-dissolved C17 (Figure 3(A); compare to Figure 2). SK-N-SH cells were also tested with RAMBA C2 packaged in liposomes (Figure 3(B)). As additional controls, empty liposomes were used, in media with or without added ATRA. Once again, liposome packaging of C2 permits the RAMBA to enhance the ATRA-induced neurite elongation by approximately 60% (*p* value .06). Preliminary data from LAN5 cells, another line that differentiates in response to ATRA, show a similar trend (not shown). Liposome-treated cells again showed that RAMBA co-treatment with ATRA enhanced the morphological shift towards highly rounded cell bodies (Figure 4; compare to Figure 2). These data collectively indicate that RAMBAs C2 and C17 can be successfully delivered to neuroblastoma cells in liposomes, where they enhance ATRA-dependent differentiation to a similar level as seen with ethanol-delivered RAMBAs (compare Figure 2 with Figure 4).

Empty liposome treatment generated no morphological effects (Figure 4). However, when combined with ATRA, we did sometimes observe an increased refractility of cells, but not as extreme as with the RAMBA liposome (Figure 4). Although not currently understood, empty liposomes may have some limited propensity to enhance ATRA, possibly by binding to RA through their positive charge or by attracting RA through its hydrophobic quality.

### Liposomal RAMBAs enhance ATRA-induced AKT signalling

**Figure 5.**
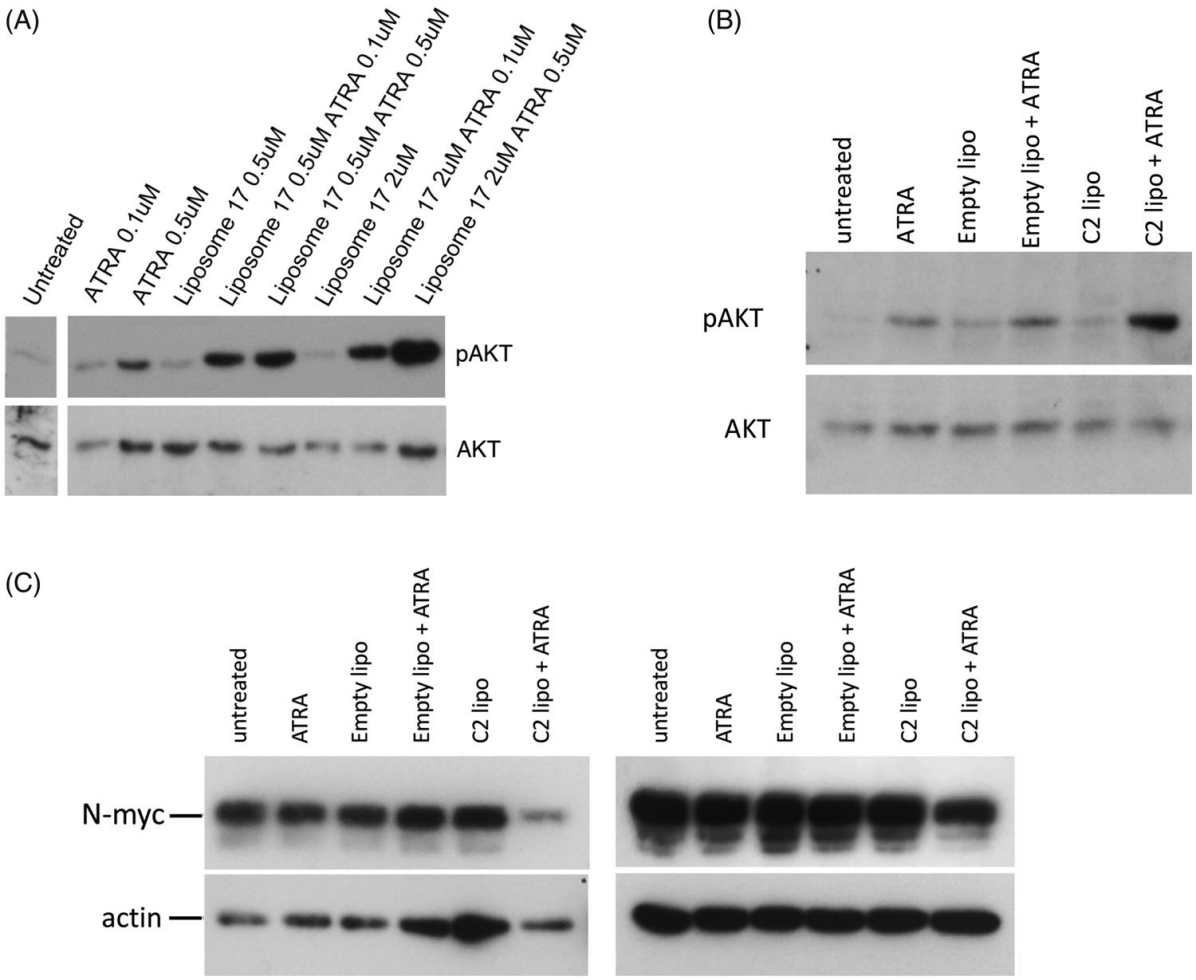
(A) SK-N-SH cells were treated for 72 h with ATRA, C17 liposomes (C17 lipo), or combinations. Immunoblotting shows levels of pAKT and AKT; the untreated lane is from the same blot. (B) A second experiment is shown in which SK-N-SH was treated with ATRA alone, or in combinations with empty liposomes or C2 liposomes (C2 lipo). Immunoblotting of pAKT and AKT is shown. (C) LAN5 cells were treated for 24 h with 0.1 µM ATRA alone and in combinations with empty liposomes and C2-containing liposomes. Immunoblotting shows levels of N-myc and actin. Two independent experiments are shown and maximal, hypothetical concentrations of the RAMBAs are indicated.

As shown in Figure 2, RAMBAs can enhance pAKT levels in the presence of ATRA. To assess whether liposomal RAMBAs retain this activity, SK-N-SH cells were treated with C17 liposomes and ATRA (Figure 5(A)). The combination treatments again enhance pAKT levels above those seen with single treatments. Further experiments were carried out with C2 liposomes, empty liposomes and lower levels of ATRA (0.05 µM) (Figure 5(B)). Again, the C2 liposomes specifically increased pAKT stimulation in combination with ATRA. This effect may be close to maximal with 0.5 µM C2, since 2 µM C2 showed a lesser stimulation of pAKT (although not statistically different from the 0.5 µM mean), possibly due to mild detrimental effects of the higher lipid treatment. pAKT activation was comparable between ethanol-delivered C2 and liposomal C2 (compare Figure 5 and Figure 2(C)). Preliminary data using C17 also show pAKT stimulation (Supplementary Figure 2).

### N-myc suppression by RAMBAs and retinoids

C2 and C17 are capable of enhancing ATRA actions as judged by pAKT and differentiation. A further outcome of retinoid treatment in neuroblastoma cells is the suppression of N-myc protein levels [39,40]. We therefore tested whether liposomal RAMBAs were capable of enhancing N-myc suppression by ATRA. The MYCN-amplified cell line LAN5 was treated with liposomes and low levels of ATRA for 24 h. Figure 5(C) shows the modest N-Myc response with ATRA at this 0.05 µM dose, but an enhanced suppression of N-Myc in cells treated with the ATRA and C2 liposome combination.

The collective data therefore indicate that RAMBAs encapsulated in liposomes have good bioavailability in neuroblastoma cells and are effective at augmenting ATRA signalling as evidenced by enhanced neurite elongation, AKT phosphorylation and N-myc suppression.

### Sustained morphological differentiation after RAMBA liposome delivery

**Figure 6.**
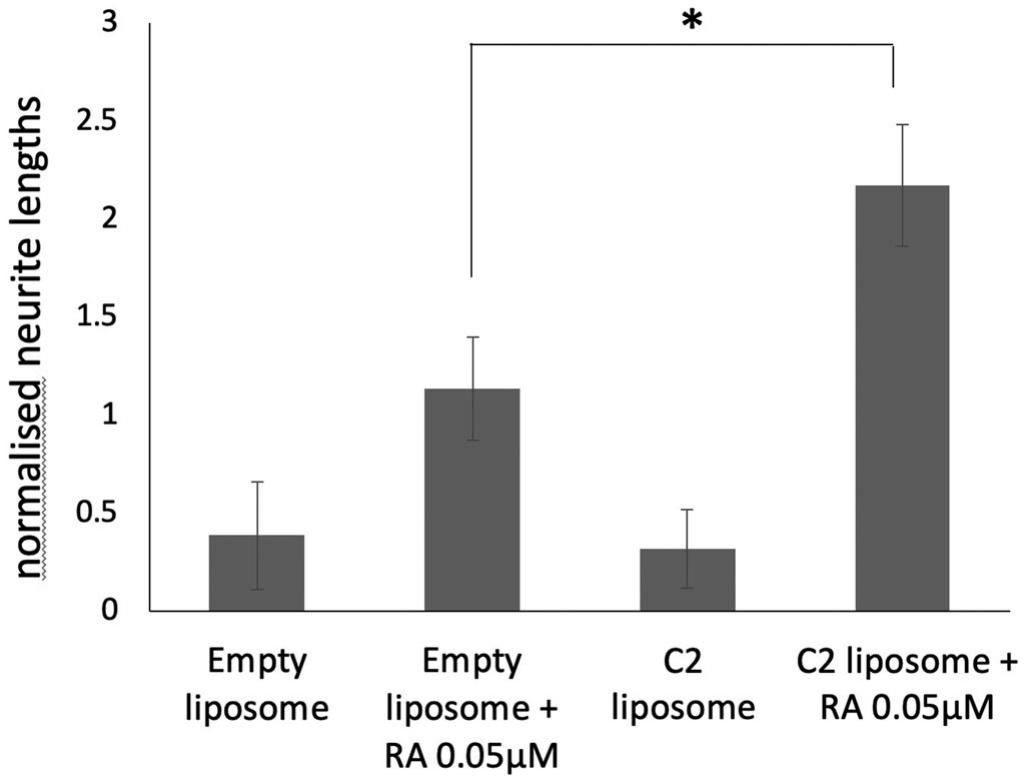
SK-N-SH cells were treated with empty liposomes or C2 liposomes for 24 h. The media was then replaced, with and without 0.05 µM ATRA, and the cells were cultured for six more days. Neurite lengths were measured and means ± SD are shown (*n* = 3). ANOVA shows significant difference *p<.05.

Previous data in the field indicate that liposomes bind to cells maximally within 4–6 h [41,42]. We wished to test if liposomal RAMBAs retained extended activity after a short cellular treatment. We treated LAN5 cells with liposomes for 24 h and this cell media was then removed and replaced with fresh media containing only ATRA. Figure 6 shows that C2 liposome pre-treatment still led to enhanced ATRA-dependent neurite elongation, six days after liposome removal. Thus, RAMBAs in liposomes can be delivered to these tumour cells and will trigger a sustained, combination response with ATRA for several days.

### ATRA suppression of cell growth is enhanced by liposomal RAMBAs

RAMBAs enhance retinoid-induced growth suppression in breast cancer cells [24,33], but the equivalent has yet to be demonstrated in neuroblastoma cells. RA increases differentiation in some neuroblastoma cell lines and in others it can reduce proliferation as well as, or instead of differentiation. We therefore tested the ability of liposomal RAMBAs to enhance the ability of ATRA to suppression cell proliferation.

**Figure 7.**
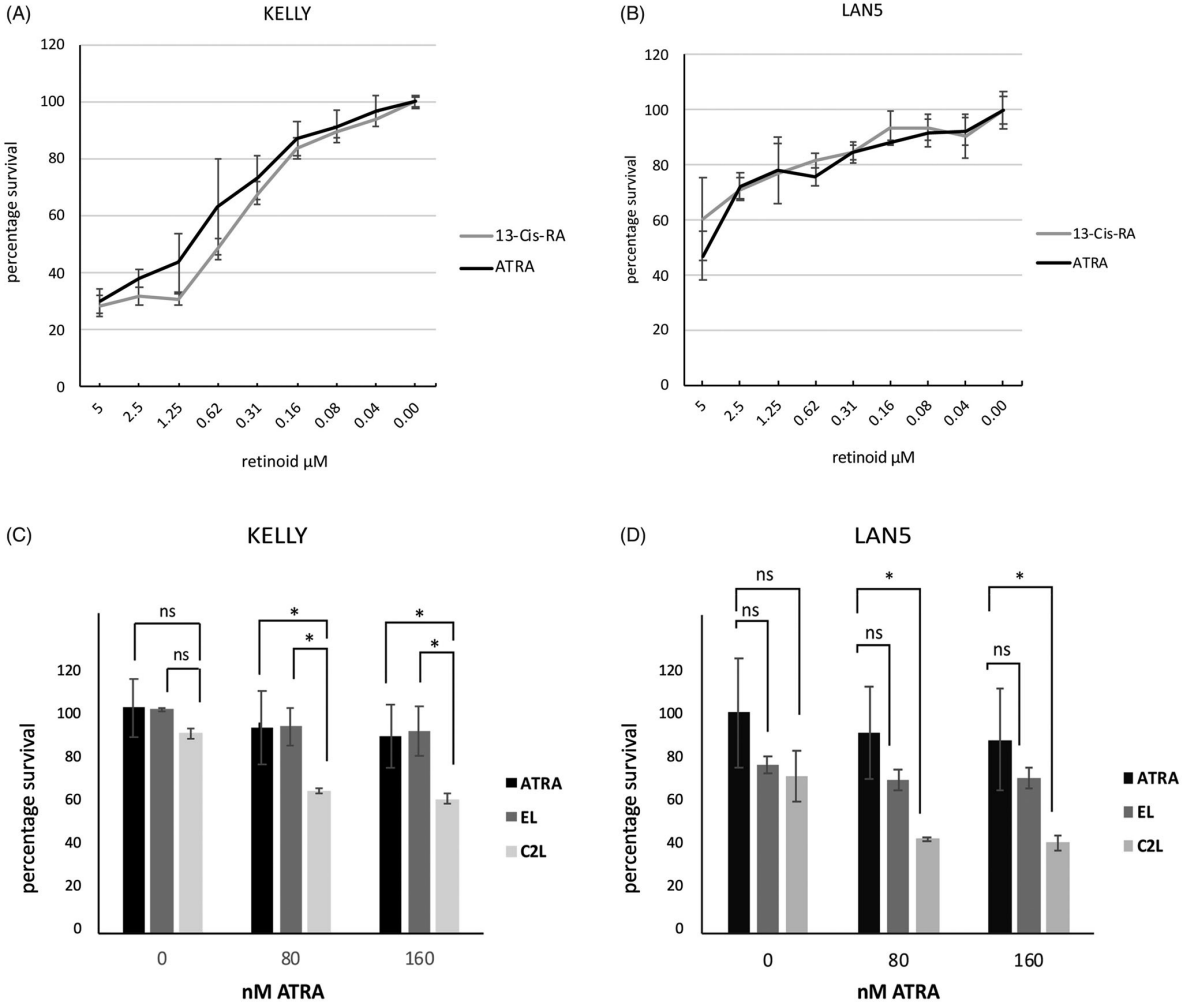
(A, B) KELLY and LAN5 cells were plated in 96-well plates and grown in the presence of a range of concentrations of ATRA or 13-cis-RA. The relative cell survival was measured after six days and plotted with means and SD (*n* = 3); zero ATRA is treated as 100% survival. (C, D) Cell proliferation rates were assessed in KELLY and LAN5 cells after treatment for six days with zero, 80 nM or 160 nM ATRA, plus either empty liposomes or C2-containing liposomes at 0.5 µM C2 (maximal concentration) (*n* = 3). ANOVA analysis was performed, and SDs are shown. *p<.05; ns: not significant (*p*>.05).

When used alone, ATRA suppressed LAN5 and KELLY cell proliferation. We also compare 13-cis-RA to ATRA, since 13-cis-RA is used clinically in neuroblastoma treatment. A broadly comparable pattern of growth suppression was observed (Figure 7(A,B)). In KELLY, the EC_50_ value for ATRA was approximately 0.6 µM, and 0.3 µM for 13-cis-RA. In LAN5, both EC_50_ values were approximately 0.3 µM. This indicates that under these conditions the two retinoids act similarly in LAN5, and in KELLY the 13-cis-RA is slightly more effective. The 13-cis-RA is thus likely isomerised efficiently into intracellular ATRA in these cells.

The cells’ responses to treatment with either empty liposomes or C2-containing liposomes were assessed. Cells were treated with 80 nM or 160 nM ATRA, well below its EC_50_, for six days, alongside either 0.5 µM C2 liposomes or the equivalent amount of empty liposomes to match the lipid dose. C2 liposomes were able to significantly suppress growth in ATRA-treated KELLY and LAN5 cells compared with minimal growth suppression by ATRA alone (Figure 7(C,D)). In ATRA-treated KELLY cells, C2 liposomal treatment suppressed growth more than the empty liposomes. In LAN5 cells, both liposome formulations somewhat suppressed cell proliferation at 0 µM ATRA, although this did not reach significance. C2 liposomes plus ATRA showed a suppression of LAN5 growth compared with ATRA alone, whereas the empty liposomes did not. At higher levels of ATRA up to 1 µM, the C2 liposomes could still suppress cell growth (Supplementary Figure 3). These data demonstrate that C2 liposomes can enhance the growth suppression driven by ATRA, corroborating what was found with neurite elongation, N-myc suppression and AKT activation.

### C2 packaging efficiency in liposomes

The experiments described so far assumed the maximal dose estimates of C2 and C17 when encapsulated in liposomes. Maximal encapsulation, however, is not routinely achieved with liposomal formulations. During the completion of our biochemical and cellular analyses, we were able to successfully develop a UV assay and this was used to assess the actual RAMBA concentrations in liposomal formulations.

**Figure 8.**
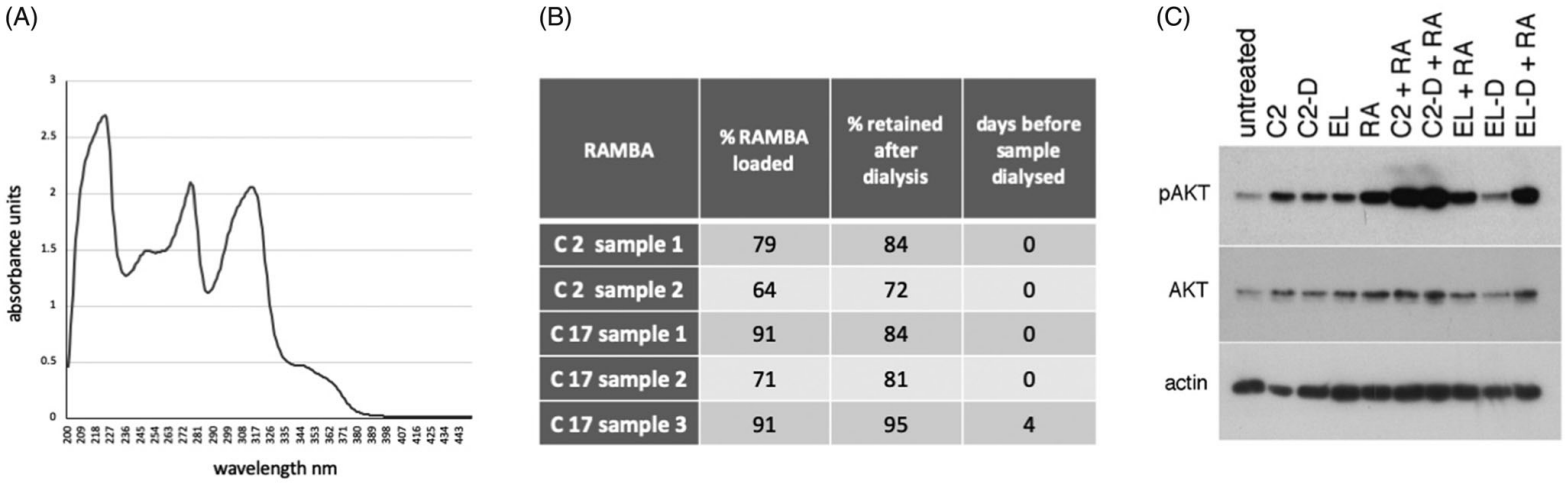
Assessment of the loading and retention of RAMBAs in liposomes. (A) A UV spectrophotometric scan of 100 µM C17 dissolved in ethanol. (B) Table showing independent preparations of liposomes tested for the amount of RAMBA present, expressed as a percentage of the theoretical maximum of 240 µM. The percentage of this RAMBA that remained after a 24 h dialysis against distilled water is given, as well as the time delay between liposome synthesis and the dialysis. (C) Immunoblot of pAKT, AKT and actin from KELLY cells that were treated for 24 h with C2-containing liposomes before (C2) or after (C2-D) dialysis, empty liposomes before (EL) or after (EL-D) dialysis, with or without co-treatment with 0.1 µM ATRA (RA).

RAMBAs were first solubilised in ethanol and UV absorbance scans were performed from 200 to 450 nm (Figure 8(A)). A peak at 315 nm was chosen for generating a standard curve since this wavelength showed low background absorbance by empty liposomes (see below). To calculate the amount of C2 and C17 present in liposomes, each batch of RAMBA liposomes was dissolved in ethanol at 10% v/v, and UV scans performed (Figure 8(B)). Empty liposomes were similarly scanned for background subtraction. The physical parameters of these particular liposomes are given in Supplementary Table 1. Compared to the maximal theoretical packaging value of 240 µM, the actual encapsulation efficiency of the RAMBAs was 64–79% for C2 and 71–91% for C17 (Figure 8(B)). Based on this, the doses used in our cellular experiments with liposomal RAMBAs could be overestimated from between 10% and 30%.

We also assessed RAMBA retention in the liposomes, by dialysing the liposomes over 24 h and quantifying the remaining RAMBA (Figure 8(B)). This showed that 72–84% of C2 remained encapsulated after dialysis, and 81–95% of C17 was retained. The C17 sample 3 was left for four days before dialysis and these still retained 95% of the original RAMBA, indicating that nearly all of the RAMBA is retained over this extended time period.

To test if the RAMBA liposomes retained activity after dialysis, we tested the ability of dialysed C2 liposomes to induce morphological differentiation and AKT activation. The dialysed liposomes retained their qualitative effects on ATRA-induced differentiation, generating highly rounded, phase bright neurons (Supplementary Figure 1). Biochemically, the dialysed liposomes also enhanced ATRA stimulation of pAKT levels similarly to their non-dialysed liposome counterparts over this shorter 24 h assay (Figure 8(C)).

## Discussion

RA has proven to be a beneficial biological therapeutic in leukaemia and neuroblastoma and in several keratinisation disorders [11,43]. Although the potential clinical benefits of retinoids are broad, there are significant, practical challenges with their systemic delivery. For paediatric diseases such as neuroblastoma in particular, dosing is physically difficult and off-target toxicity is well documented [44,45]. ATRA and 13-cis-RA also have poor pharmacokinetics and are metabolised *in vivo* by induced CYP26 enzymes [2,6]. Lastly, retinoid resistance often develops in cancer patients, being caused in part by sustained upregulation of the same CYP26 enzymes [7–9,46]. Given the relatively modest clinical benefit of current RA treatment in neuroblastoma [2], there is a continued need to understand whether there is greater efficacy still to be gained and where the long-term potential lies.

Interest in RAMBAs in recent years has focussed on whether these RA metabolism blockers could be practicable tools for enhancing RA efficacy and reducing resistance. RAMBAs may also increase the intracellular activity of RA without significantly increasing its systemic toxicity. Previous work by Armstrong et al. had shown that the CYP26 inhibitor R116010, co-applied with 13-cis-RA in animals, reduces 13-cis-RA metabolism in the liver, leading to higher levels of serum 13-cis-RA and ATRA, plus greater *CYP26A1* transcription in xenografted SH-SY5Y tumours [18]. That study demonstrated that CYP26-specific RAMBAs sustained increased 13-cis-RA levels in the blood, showing a systemic benefit. Extrapolation to humans is uncertain, however, since the specificity of R116010 towards murine CYP enzymes was questioned. Although R116010 has also been shown to suppress breast cancer cell growth [47], this drug was not developed further and clinical trials did not progress. Instead of R116010, we used RAMABA C2 and C17 here to assess RAMBA potential in cultured neuroblastoma cells. C2 has a similar IC_50_ to R116010 (3 nM), whereas the IC_50_ of C17 is 10-fold lower (0.35 nM) [19]. These RAMBAs could therefore be very effective CYP26 inhibitors for use *in vivo*, but only if they can be delivered at effective doses. However, their high hydrophobicity presents a barrier to this, and we have addressed this in this study by testing their delivery to cells using liposome nanotechnology.

RAMBAs C2 and C17 delivered after ethanol solubilisation alongside ATRA, induced high levels of *CYP26A1* mRNA transcription in SK-N-SH cells, agreeing with previous studies [19]. We then demonstrated for the first time that C2 and C17, when dissolved in ethanol and combined with ATRA, could stimulate ATRA-dependent neuronal differentiation as judged by increased neurite elongation, and increased pAKT activation. These RAMBAs can therefore enhance ATRA action at a morphological and biochemical level in these tumour cells. To our knowledge, RAMBAs have not be delivered using nanotechnology before and we have therefore provided the first demonstration that these hydrophobic molecules can be incorporated effectively into liposomes. In such a context, they retain bioavailability and an activity that is at least comparable to that seen with their ethanol-dissolved counterparts. These liposomal RAMBAs enhanced ATRA-driven neurite extension and AKT activation in SK-N-SH cells, led to greater suppression of N-myc protein and enhanced the growth-suppressive properties of ATRA. Collectively, these data demonstrate that CYP26-specific, hydrophobic RAMBAs can be effectively incorporated into liposomes where their bioavailability remains effective at enhancing ATRA actions in neuroblastoma-derived cell lines.

These effects of liposomal RAMBAs were achieved using combined retinoid concentrations as low as 50–80 nM in KELLY and IMR32 cells, a level that is readily achievable clinically [48,49]. The serum levels of 13-cis-RA given to children in current dosing regimens peaks at a mean of 2.8 µM [48] or 6.9 µM [49], depending on the study, but then drops rapidly to sub-micromolar levels within 6 h. This raises the possibility that co-treatment with liposomal RAMBAs such as C2 or C17 may be able to prolong the effective, active dose of retinoids in cells, or may even allow lower, less toxic retinoid doses to be used *in vivo*. Of perhaps more relevance, the liposomal approach, if targeted correctly, could increase the local action of retinoids while minimising off-target toxicities in normal tissues. Furthermore, our sequential treatment of cells with liposomes then with ATRA, indicated that a single liposomal delivery can generate a sustained enhancement of ATRA-induced differentiation over several days. This could be of clinical relevance if translated to a similar effect *in vivo*, as an infrequent dose of RAMBA nanocomplexes may enhance and prolong the action of the currently used, daily retinoid treatment. This warrants pre-clinical studies *in vivo* in order to test this hypothesis.

One question with RAMBAs is what their best target *in vivo* would be. For example, should they be targeted to the liver, where there is significant CYP26 induction, or to the tumours tissues? Our study was done in tissue culture, focussing on the tumour-derived cells themselves, where the RAMBAs are certainly effective. Their effectiveness systemically *in vivo* is more difficult to predict at this stage. Retinoids are efficiently destroyed in the liver and so it could be argued that RAMBAs should be targeted there in order to maintain systemic retinoid levels. Nevertheless, it may be equally effective to target the solid tumours themselves directly. The possibility of delivering RAMBAs alongside RA in combined liposome formulations should also be considered.

Although the liposomes employed in our study are conventional in design and suitable for *in vitro* experiments, further modifications will be required for optimal targeting *in vivo* [27,50]. These modifications could include alterations in concentration of the neutral lipid cholesterol to reduce toxicity, or incorporation of polyethylene glycol (PEG), which reduces aggregations and binding of serum proteins. Targeting peptides should also be considered, for directed uptake into neuroblastoma tumours, as demonstrated in xenografts [51,52], where they can extravasate and concentrate in the tumour, potentially using the enhanced permeation and retention effect [53]. There are numerous further liposome formulations in the clinic or in trials, with a range of biophysical and biochemical properties [25,27,28], so optimised tumour cell targeting should be achievable. Improved targeting may again facilitate the use of lower systemic doses of RA in children.

We noted that our empty liposomes appeared to sometimes weakly enhance the effects of ATRA. The liposomes’ positive charge could possibly play a role in adsorbing ATRA under some circumstances and future studies could evaluate if neutral or anionic liposomes have similar effects. As an alternative approach, we would also consider co-incorporation of RAMBAs and RAs into cationic liposomes for co-delivery *in vivo*. This may further reduce off-target toxicities of the retinoids.

In conclusion, hydrophobic RAMBAs derived from imidazole and triazole scaffolds with high specificity for CYP26 enzymes, can be delivered successfully to neuroblastoma cells in liposomes. They enhance the cellular and biochemical actions of RA in SK-N-SH, LAN5, IMR32 and KELLY cells. Not all neuroblastoma tumour cells however will induce CYP26 in response to RA [18] and so, as with most drugs approaches, the benefits of RAMBAs may be restricted to RA-responsive neuroblastoma tumours and resistant tumours where resistance is CYP26 enzyme-based. Use of nanotechnology to deliver RAMBAs could also be considered for applications in other cancers such as basal cell carcinoma [54], lymphoid malignancies [55] and others, where retinoids are either used clinically or show promise from pre-clinical models [10]. This study has provided a proof of principle and the basis for further investigation of nanotechnology as a delivery pathway for hydrophobic RAMBAs in combination with retinoids, with the goal of increasing the efficacy of retinoid actions in target tissues and improving clinical outcomes.

## Supplementary Material

supplementary_table_1.docx

Supplementary_Figure_4.pdf

supplementarty_figure_3.tiff

supp_figure_2.jpg

supplementray_Figure_1.jpg
