# Supplementary material for: Liposomal delivery of hydrophobic RAMBAs provides good bioavailability and significant enhancement of retinoic acid signalling in neuroblastoma tumour cells
Source: J Drug Target. 2020 Jan 14;28(6):643–54. doi: 10.1080/1061186X.2019.1710157 (PMC7609071; doi:10.1080/1061186X.2019.1710157)
Supplement: supplementary_table_1.docx [file IDRT_A_1710157_SM4254.docx]

**Supplementary Table 1. Parameters of liposomes used for RAMBA loading studies**

| **parameter** | **C2 Liposome**  **batch 1** | **C2 Liposome**  **batch 2** | **C17 Liposome**  **batch 1** | **C17 Liposome**  **batch 2** | **C17 Liposome**  **batch 3** |
| --- | --- | --- | --- | --- | --- |
| **size** | 200 | 191 | 321 | 390 | 175 |
| **PDI** | 0.38 | 0.55 | 0.49 | 0.41 | 0.53 |
| **charge** | 58.3 | 59.4 | 70.6 | 74.2 | 58.4 |
