# Supplementary figures and images for: Liposomal delivery of hydrophobic RAMBAs provides good bioavailability and significant enhancement of retinoic acid signalling in neuroblastoma tumour cells
Source: J Drug Target. 2020 Jan 14;28(6):643–54. doi: 10.1080/1061186X.2019.1710157 (PMC7609071; doi:10.1080/1061186X.2019.1710157)

## Supplementary Figure 4

A

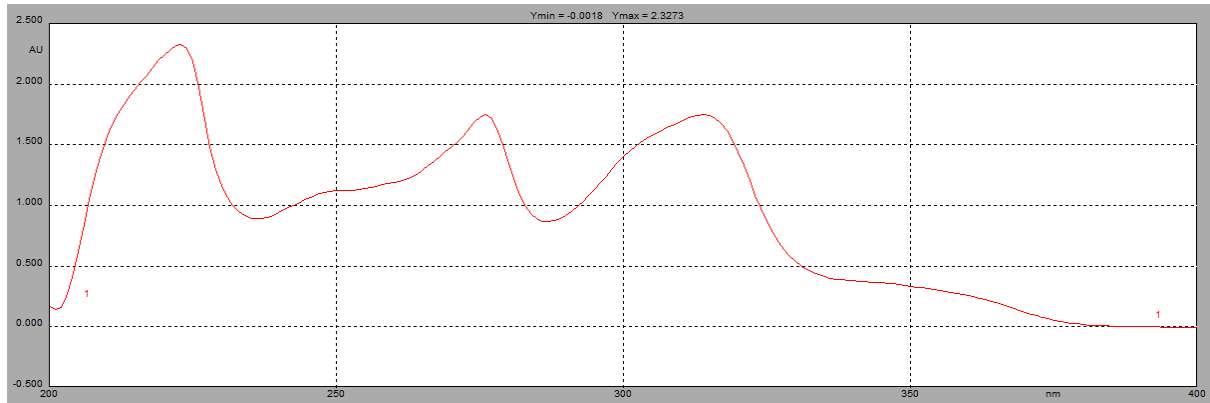

B

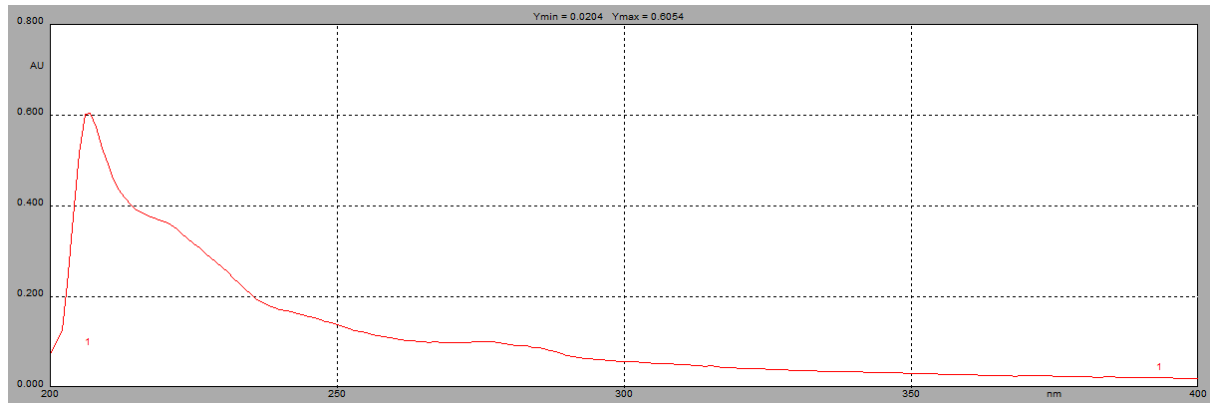

C

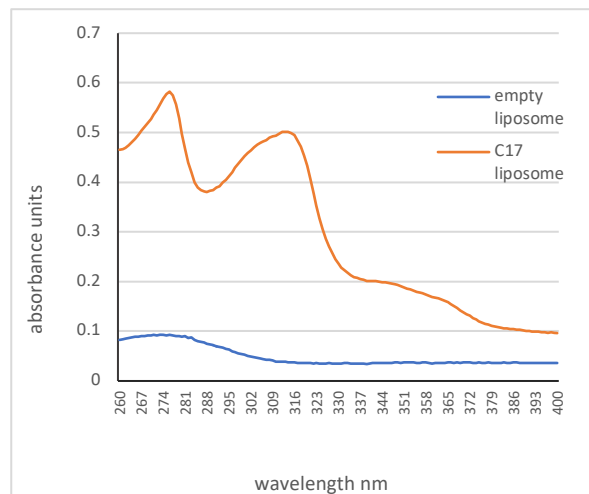

Supplement: Supplementary_Figure_4.pdf [file IDRT_A_1710157_SM4253.pdf]

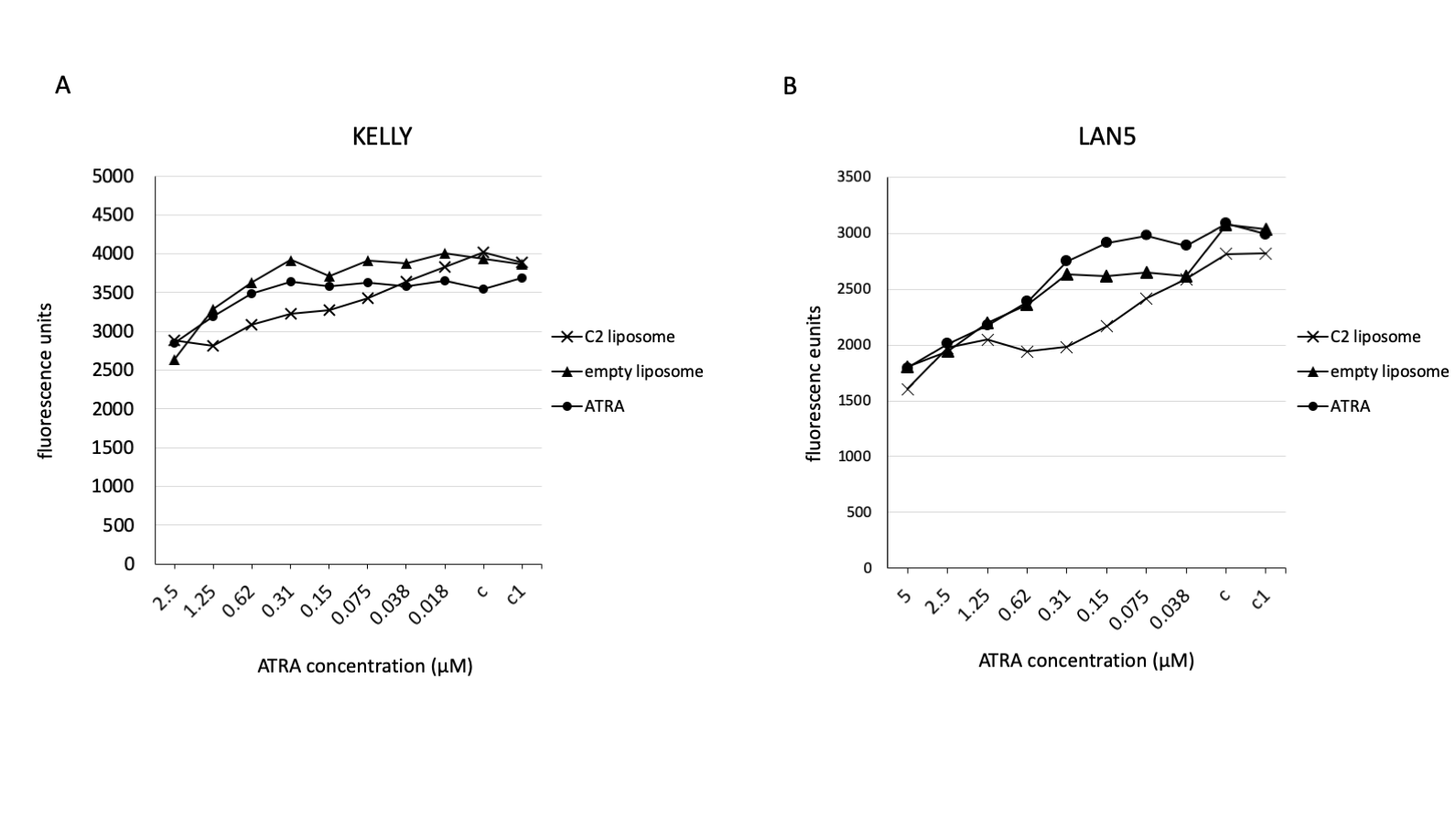

Supplement: supplementarty_figure_3.tiff [file IDRT_A_1710157_SM4252.tiff]

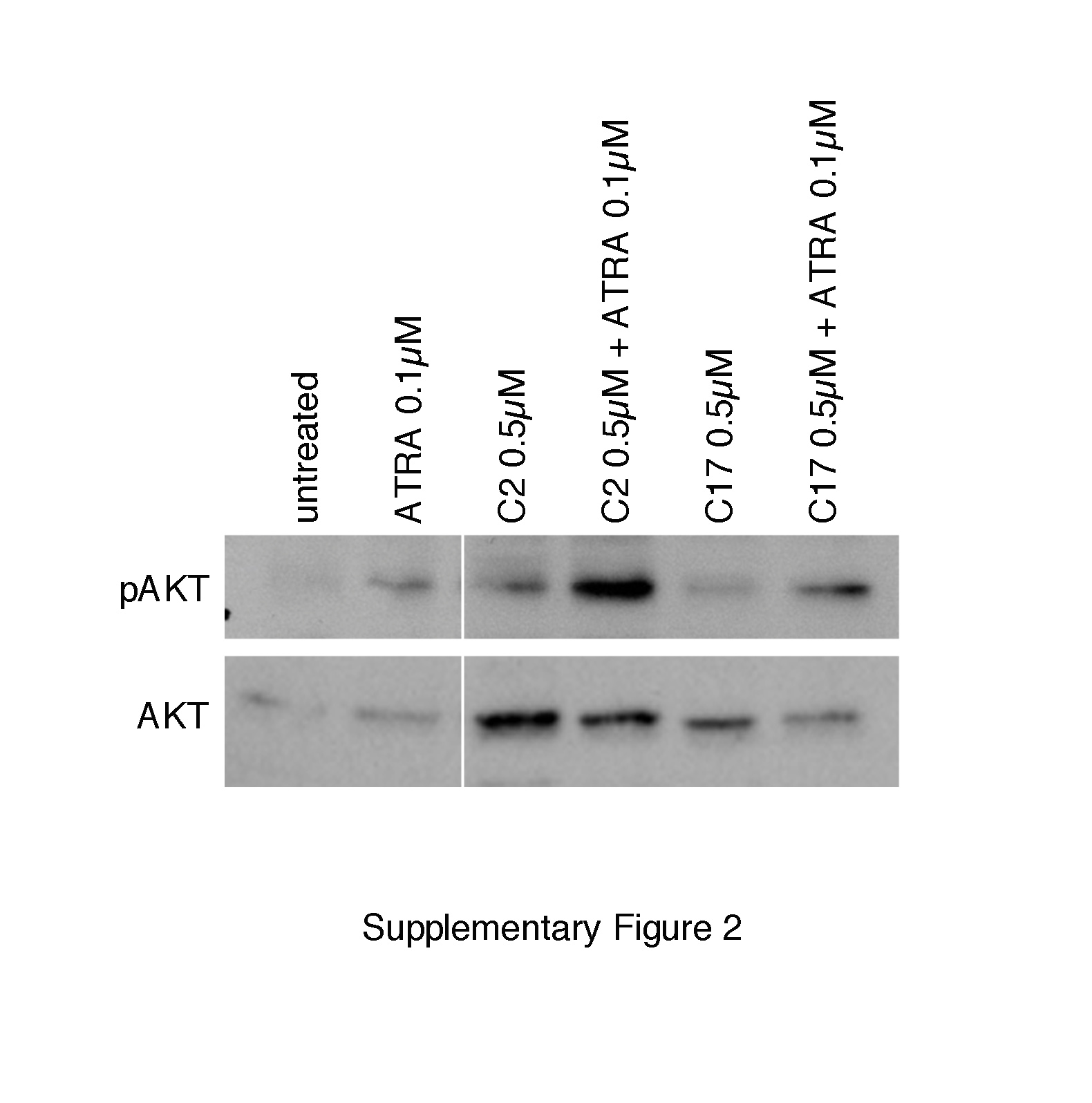

Supplement: supp_figure_2.jpg [file IDRT_A_1710157_SM4251.jpg]

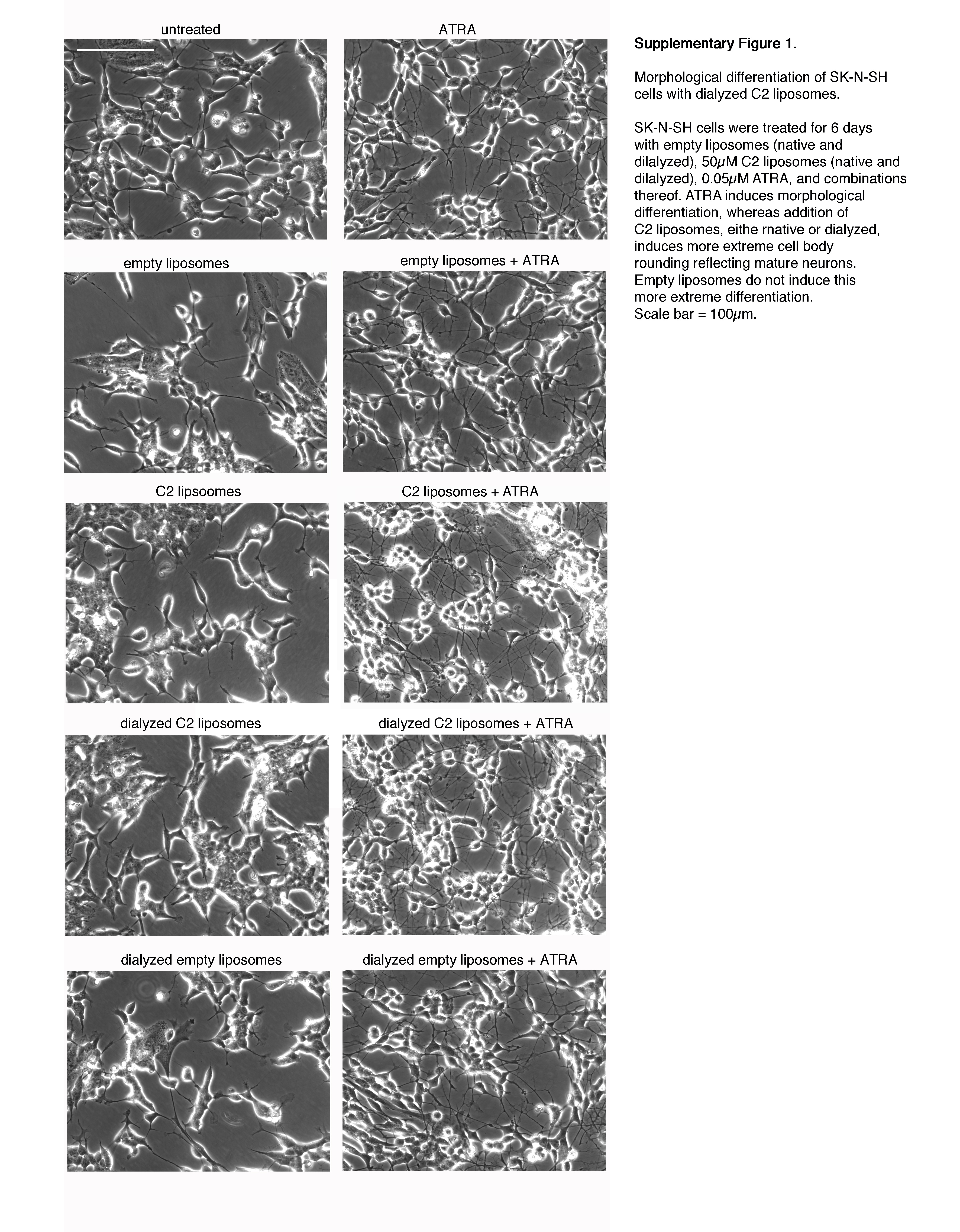

Supplement: supplementray_Figure_1.jpg [file IDRT_A_1710157_SM4250.jpg]
